# Outcomes of Induction Chemotherapy for Head and Neck Cancer Patients: A Combined Study of Two National Cohorts in Taiwan

**DOI:** 10.1097/MD.0000000000002845

**Published:** 2016-02-18

**Authors:** Jin-Hua Chen, Yu-Chun Yen, Shing-Hwa Liu, Sheng-Po Yuan, Li-Li Wu, Fei-Peng Lee, Kuan-Chou Lin, Ming-Tang Lai, Chia-Che Wu, Tsung-Ming Chen, Chia-Lun Chang, Jyh-Ming Chow, Yi-Fang Ding, Ming-Chin Lin, Szu-Yuan Wu

**Affiliations:** From the Biostatistics Center and School of Public Health, Taipei Medical University (J-HC, Y-CY); Institute of Toxicology, College of Medicine, National Taiwan University (S-HL, S-YW); Department of Otorhinolaryngology (S-PY, F-PL, M-TL, C-CW, T-MC, Y-FD); Department of Oral and Maxillofacial Surgery (K-CL); Department of Hemato-Oncology (C-LC, J-MC); Department of Radiation Oncology, Wan Fang Hospital (S-YW); Department of Internal Medicine, School of Medicine, College of Medicine, Taipei Medical University, Taipei (J-MC, S-YW); Department of Biotechnology, Hungkuang University, Taichung (S-YW); Department of Ophthalmology, Buddhist Tzu Chi General Hospital (LLW); and Graduate Institute of Biomedical Informatics, Taipei Medical University, Taipei, Taiwan, R.O.C. (S-PY, M-CL); Department of Neurosurgery, Taipei Medical University-Shuang Ho Hospital, Taipei, Taiwan (M-CL).

## Abstract

The use of induction chemotherapy (CT) is controversial. We compared the survival of head and neck cancer patients receiving docetaxel- or platinum-based induction CT before concomitant chemoradiotherapy (CCRT) with the survival of those receiving upfront CCRT alone.

Data from the National Health Insurance and cancer registry databases in Taiwan were linked and analyzed. We enrolled patients who had head and neck cancer between January 1, 2002 and December 31, 2011. Follow-up was from the index date to December 31, 2013. We included head and neck patients diagnosed according to the International Classification of Diseases, Ninth Revision, Clinical Modification codes 140.0–148.9 who were aged >20 years, at American Joint Committee on Cancer clinical cancer stage III or IV, and receiving induction CT or platinum-based CCRT. The exclusion criteria were a cancer history before head and neck cancer diagnosis, distant metastasis, AJCC clinical cancer stage I or II, receipt of platinum and docetaxel before radiotherapy, an age <20 years, missing sex data, docetaxel use during or after RT, induction CT for >8 weeks before RT, induction CT alone before RT, cetuximab use, adjuvant CT within 90 days after RT completion, an RT dose <7000 cGy, curative head and neck cancer surgery before RT, nasopharyngeal cancer, in situ carcinoma, sarcoma, and head and neck cancer recurrence.

We enrolled 10,721 stage III–IV head and neck cancer patients, with a median follow-up of 4.18 years (interquartile range, 3.25 years). The CCRT (arm 1), docetaxel-based induction CT (arm 2), and platinum-based CCRT (arm 3; control arm) groups comprised 7968, 503, and 2232 patients, respectively. Arm 3 was used to investigate mortality risk after induction CT. After adjustment for age, sex, clinical stage, and comorbidities, the adjusted hazard ratios (aHRs) (95% confidence interval [CI]) for overall death were 1.37 (1.22–1.53) and 1.44 (1.36–1.52) in arms 2 and 3, respectively. In a disease-specific survival rate analysis, aHRs (95% CI) of head and neck cancer-related death were 1.29 (1.14–1.46) and 1.47 (1.38–1.56) in arms 2 and 3, respectively.

Compared with CCRT alone, docetaxal- or platinum-based induction CT did not improve survival but increased the risk of all-cause and head and neck cancer-related death.

## Abstract 2

Supplemental Digital Content is available in the text

## INTRODUCTION

Head and neck cancers are the 6th most common cancer worldwide, with approximately 650,000 cases of and 200,000 deaths associated with head and neck cancers occurring annually. In the United States, approximately 54,000 new head and neck cancer cases are diagnosed annually.^1^ In certain parts of Asia (eg, Taiwan), head and neck cancers are the 4th leading cause of cancer death and the 6th most common cancer.^2^ Unresectable locoregional disease (stages III–IV) is amenable to multimodality therapy – typically a combination of chemotherapy (CT) and radiation therapy (RT). However, the 5-year survival rate after multimodality therapy remains poor (<50%);^3^ therefore, effective treatments are required.

The use of induction CT is controversial. In a large-scale meta-analysis, concomitant chemoradiotherapy (CCRT) was established as the standard treatment for locally advanced head and neck squamous cell carcinoma.^4^ However, less than 50% of the study patients survived after 3 years of treatment; therefore, alternative treatment strategies are being explored. In addition, a 5-year survival advantage was observed when patients underwent platinum- and 5-fluorouracil (5-FU)-based induction CT (hazard ratio [HR] 0.9, 95% confidence interval [CI] 0.82–0.99).^4^ Although this advantage was slight, it was sufficient to warrant further research on induction CT regimens.

In 2007, docetaxel emerged as a novel active compound in 2 large-scale randomized trials – TAX 323 and TAX 324 – in which the docetaxel-based induction regimens were more effective than platinum-based induction regimens were. In both trials, only patients responding well to treatment were selected and subjected to heavy 6-month sequential induction CT followed by CCRT.^5,6^ Nevertheless, the 3-year overall survival rate was 37% to 62% and 26% to 48% in the docetaxel and platinum groups, respectively. Thus, the use of induction regimens had no substantial survival benefits compared with CCRT treatment alone.

Similarly, 2 other phase III trials, the PARADIGM trial by the Dana-Farber Cancer Institute and the DECIDE trial by the University of Chicago^7,8^ compared the clinical outcomes of patients receiving docetaxel-based induction CT before CCRT with those of patients receiving upfront CCRT alone. Although both trials were closed to accrual, neither fulfilled the intended accrual targets; the final data from these trials are not yet available. In the present study, we studied the treatment outcomes through a national cohort study and determined whether induction therapy plus full-dose CCRT can improve survival. Furthermore, we compared the survival rates in patients receiving docetaxel- or platinum-based induction CT before CCRT with those of patients receiving upfront CCRT alone.

## PATIENTS AND METHODS

Data from the Taiwan National Health Insurance (NHI) and cancer registry databases were linked for the analysis, and 2 cohorts were formed. These 2 databases cover approximately 99% of the population of Taiwan. Patients diagnosed with head and neck cancer from January 1, 2002 to December 31, 2011 were enrolled. The follow-up duration was considered from the index date to December 31, 2013. The Taiwan NHI Bureau releases research-oriented datasets through the Collaboration Center of Health Information Application (CCHIA); these datasets include all the original claims data and registration files of beneficiaries enrolled in the CCHIA. Taiwan launched the CCHIA program in 1995, and as of 2015, it covered 99% of the population of Taiwan. Thus, the CCHIA enables researchers to trace all uses of medical services for all head and neck cancer patients in Taiwan. Abundant cancer-related information, including clinical stages, treatment modalities, pathological data, CT regimens, and CCRT or sequential CRT and RT doses, is available in the cancer registry database. Before accessing the datasets, researchers must sign an agreement contract for protecting patient information. Researchers can access the CCHIA database to analyze specific topics only. Patient identification numbers in the data sets are encrypted, completely preventing patient identification. In this study, the diagnoses of the enrolled patients were confirmed according to their pathological data, and new head and neck cancer patients had no other cancer or distant metastasis. The inclusion criteria were as head and neck cancer (identified according to the International Classification of Diseases, Ninth Revision, Clinical Modification [ICD-9-CM]codes 140.0–148.9), an age >20 years, American Joint Committee on Cancer (AJCC) clinical cancer stage III or IV (locally advanced head and neck cancers without metastasis), and receipt of induction CT or platinum-based CCRT. The index date was the date of the first head and neck cancer diagnosis. The exclusion criteria were a history of cancer before diagnosis of head and neck cancer, distant metastasis, AJCC clinical cancer stage I or II, missing sex data, an age <20 years, platinum- and docetaxel-based therapy before RT, docetaxel use during or after RT, induction CT for >8 weeks before RT, only 1 course of induction CT before RT, cetuximab use, adjuvant CT within 90 days after RT completion, an RT dose <7000 cGy, curative head and neck cancer surgery before RT, nasopharyngeal cancer, in situ carcinoma, sarcoma, and head and neck cancer recurrence. In total, 30,990 head and neck cancer patients were included. We categorized the treatment modalities into arms to compare their outcomes: arm 1 denoted CCRT, arm 2 denoted docetaxel-based induction CT and comprised 3 subgroups – induction CT followed by CCRT (docetaxel + CCRT, arm 2–1), induction CT followed by RT alone (docetaxel + RT, arm 2–2), and induction CT alone (docetaxel alone, arm 2–3), and arm 3 denoted platinum-based induction CT and comprised 3 subgroups – induction CT followed by CCRT (platinum + CCRT, arm 3–1), induction CT followed by RT alone (platinum + RT, arm 3–2), and induction CT alone (platinum alone, arm 3–3). The endpoint was the death rate among the treatment modalities, and the CCRT group functioned as the control arm.

Potential confounding comorbidities were identified according to the scores of the Charlson comorbidity index (CCI), a scoring system for common comorbidities weighted according to the mortality risk.^9^ Only comorbidities observed 6 months before and after the index date were included, according to the main ICD-9-CM diagnosis codes for the 1st admission or more than 2 repeated main diagnosis codes for outpatient treatment. Age, sex, CCI score, and AJCC clinical cancer stage were controlled or used for stratification during statistical analyses.

The cumulative incidence of death was estimated using the Kaplan–Meier method, and the differences among the treatment modalities were compared using the log-rank test. After adjustment for confounders, the Cox proportional method was used for modeling the time from the index date to all-cause as well as head and neck cancer-related death among patients who received the treatments. In the multivariate analysis, HRs were adjusted for age, sex, CCI score, and clinical stage. A stratified analysis was performed to evaluate mortality risk among the treatment modalities for oral cavity (highly prevalent in Asia) and nonoral cavity cancers. All statistical analyses were performed using SAS Version 9.3 (SAS, Cary, NC); a 2-tailed *P* value <0.05 was considered statistically significant.

We focused on the effect of docetaxel- and platinum-based induction CTs on head and neck cancer patients. The HRs of these 2 induction CTs were used to determine their effectiveness. After adjustment for covariates, individual HRs were 1.32 and 1.42 (Supplemental Table 1). The type I error rate was 0.05, and the related parameters are presented in Supplemental Table 1. The power of the calculations was >0.99. We referred to Sample Size Calculations in Clinical Research for calculations.^10^

## RESULTS

We enrolled 10,721 stage III or IV head and neck cancer patients without distant metastasis, and the median follow-up duration was 4.18 years (interquartile range, 3.25 years). The CCRT (arm 1), docetaxel-based induction CT (arm 2), and platinum-based induction CT (arm 3) groups comprised 7968, 503, and 2232 patients, respectively (Table 1). The aforementioned variables were similar in all arms, with the highest CCI score and proportion of nonoral cavity cancers in arms 1 and 2, respectively. The endpoints were predominantly high for all-cause mortality and head and neck cancer-related mortality per 100 person-years in the non-CCRT groups. Incidences of head and neck death per 100 person-years were 16.03, 27.77, and 23.98 in arms 1, 2, and 3, respectively. Furthermore, overall mortality rates per person-year were 18.89, 33.43, and 27.54 in arms 1, 2, and 3, respectively.

**TABLE 1:**
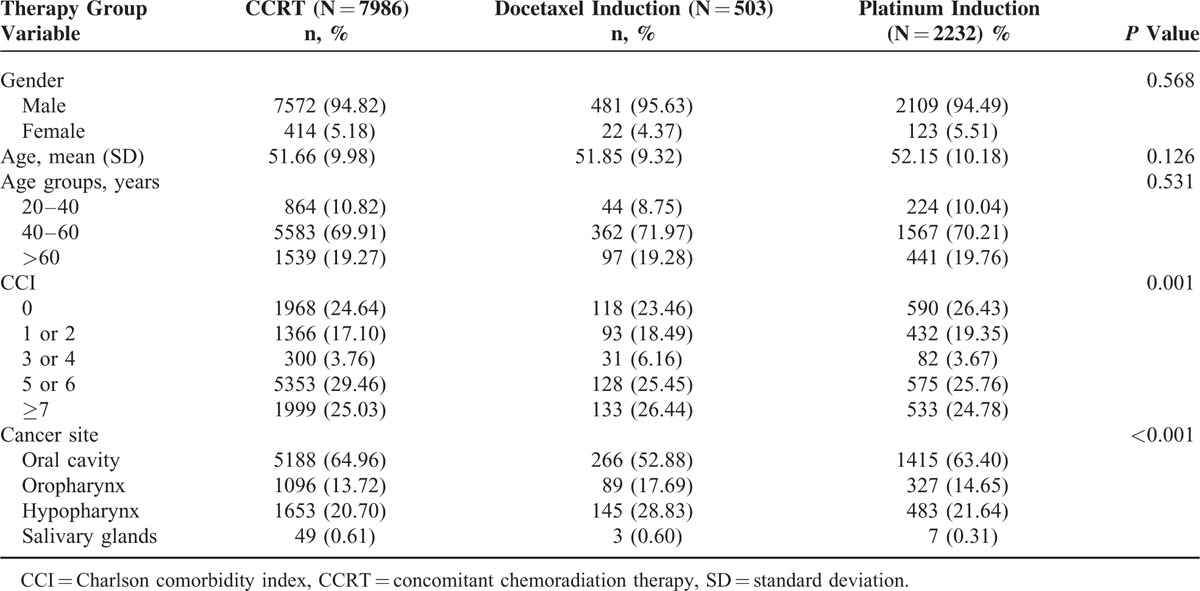
Baseline Data

**FIGURE 1:**
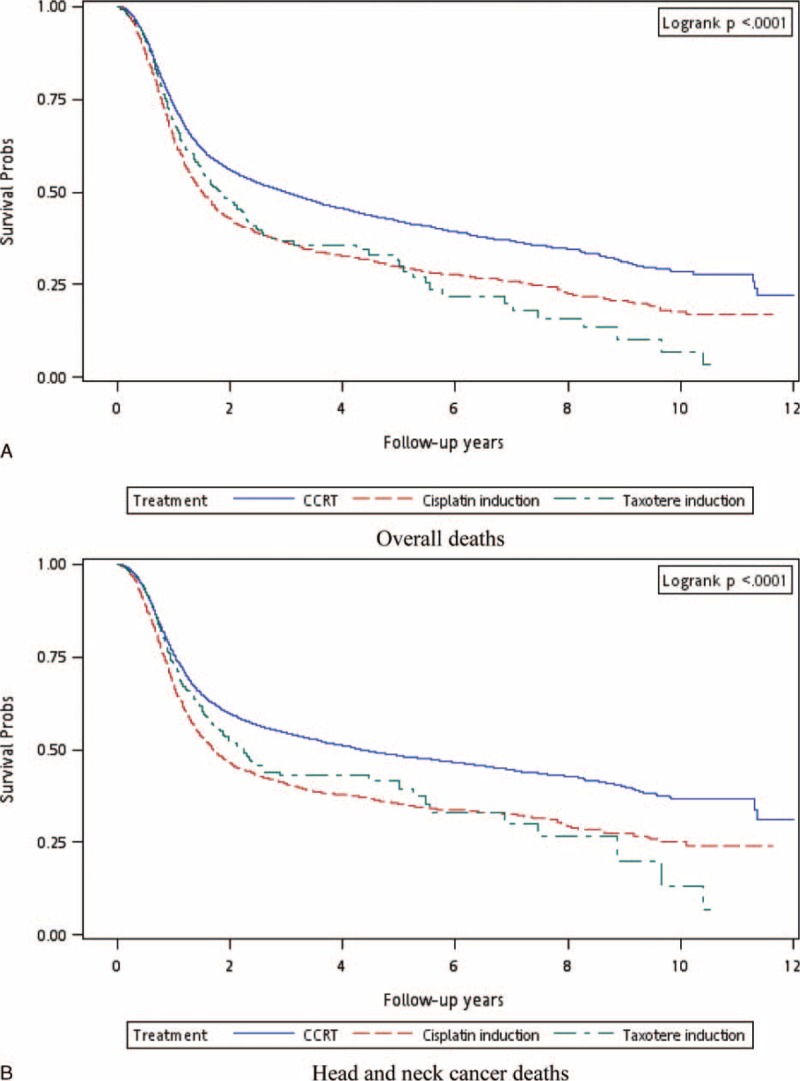
Observed endpoints. (A) All-cause and (B) head and neck cancer-related death.

The CCRT arm (arm 3) functioned as the control arm for investigating the mortality risk after induction CT. An intention-to-treat (ITT) analysis of study results is based on the initial, but not the subsequent, treatments. Here, we reasonably assumed that all head and neck cancer patients without distant metastasis status should receive RT within 4 to 8 weeks of completing CT. If the head and neck cancer patients without distant metastasis received only induction CT, they may have dropped out after receiving CT. The ITT approach enables unbiased comparisons among treatment groups and is used to eliminate the effects of crossing over and dropping out, which may alter the effects of initially administered treatment. This approach also provides information on the potential effects of the treatment approach, rather than those of specific treatments. With this rationale, we included induction CT alone in our analysis. The dropout rates in the docetaxel- and platinum-based induction CT groups were 9.94% and 21.90%, respectively (Table 2). After adjustment for age, sex, clinical stage, and comorbidities, the adjusted HRs (aHRs) (95% CI) for overall death were 1.37 (1.22–1.53) and 1.44 (1.36–1.52) in arms 2 and 3, respectively. The aHRs (95% CI) for overall death were 1.30 (1.14–1.48), 1.35 (1.00–1.82), and 2.11 (1.53–2.91) in arms 2–1, 2–2, and 2–3, respectively. The aHRs (95% CI) for overall death were 1.32 (1.23–1.42), 1.38 (1.23–1.55), and 1.90 (1.71–2.11) in arms 3–1, 3–2, and 3–3, respectively. Figure 1A shows the overall survival curve for the three treatment arms. The highest overall survival rate was observed in the CCRT group (log-rank test, *P* < 0.0001) and appeared unrelated to the initiation of different treatments. Table 3 lists the results of the stratified analyses of all-cause mortality risk in oral cavity and nonoral cavity cancers. The aHRs for all-cause mortality at the 2 anatomical sites were similar. Patients who received docetaxel- and platinum-based induction CT had poor overall survival compared with those who received CCRT. The overall survival for nonoral cavity cancers between the docetaxel + CCRT and CCRT groups was not significant. The mortality risk for nonoral cavity cancers was higher in patients who received induction CT compared with those who received CCRT, and the aHRs (95% CI) for all-cause death were 3.87 (2.24–6.69) and 4.22 (3.39–5.25) in arms 2–3 and 3–3, respectively.

**TABLE 2:**
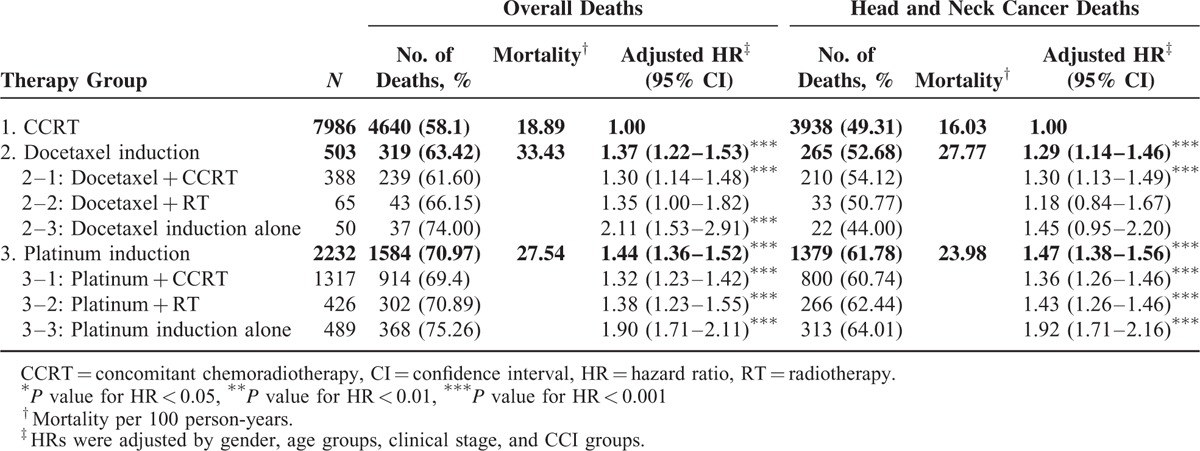
Observed Endpoints and Results of the Cox Regression

**TABLE 3:**
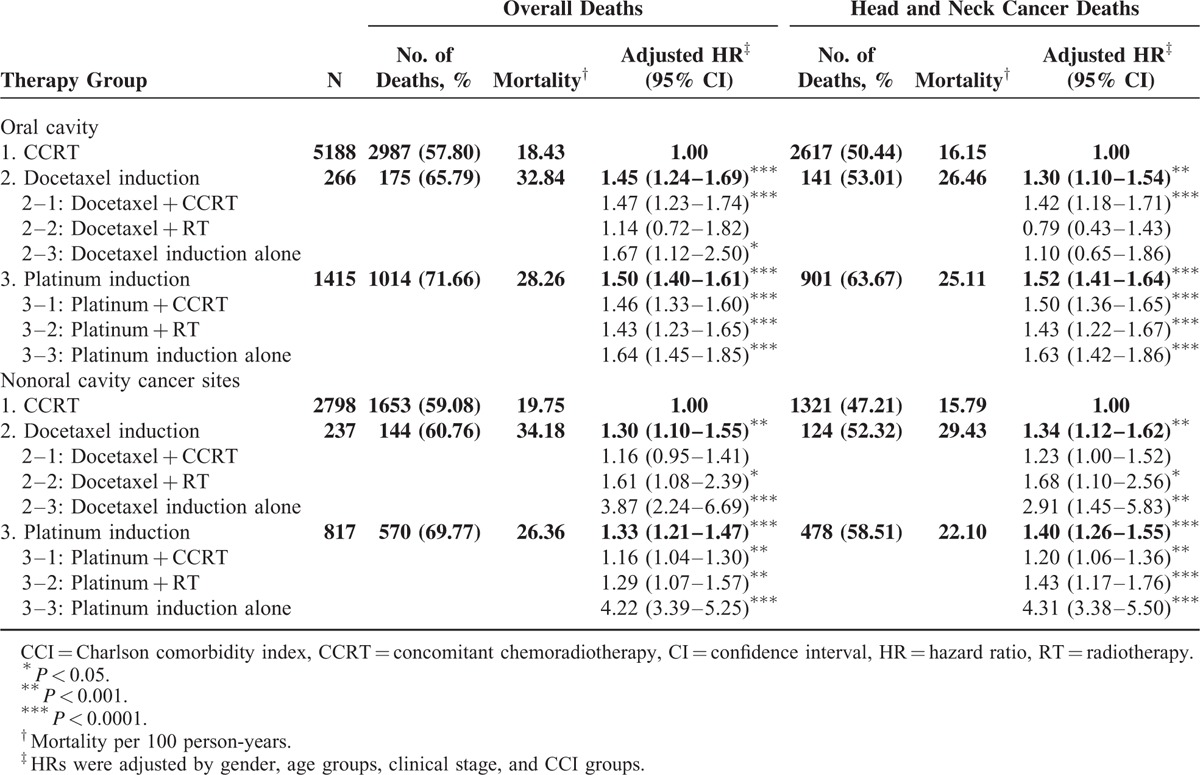
Observed Endpoints and Results of the Cox Regression Stratified by Cancer Site

After adjustment for age, sex, clinical stage, and comorbidities, the aHRs (95% CI) for head and neck cancer-related death were 1.29 (1.14–1.46) and 1.47 (1.38–1.56) in arms 2 and 3, respectively (Table 3). Furthermore, the aHRs (95% CI) for head and neck cancer-related death were 1.30 (1.13–1.49), 1.18 (0.84–1.67), and 1.45 (0.95–2.20) in arms 2–1, 2–2, and 2–3, respectively. The aHRs (95% CI) for head and neck cancer-related death were 1.36 (1.26–1.46), 1.43 (1.26–1.46), and 1.92 (1.71–2.16) in arms 3–1, 3–2, and 3–3, respectively. Figure 1B shows the disease-specific survival curve for the 3 treatment arms; the highest disease-specific survival rate was observed in the CCRT group (log-rank test, *P* < 0.0001). Table 3 lists the results of a stratified analysis of head and neck cancer-related mortality risk in oral and nonoral cavity cancers. The aHRs for overall death at the 2 anatomical sites were similar. Patients who received docetaxel- or platinum-based induction CT had poor disease-specific survival compared with those who received CCRT. The disease-specific survival in nonoral cavity cancers did not significantly differ between the docetaxel + CCRT and CCRT groups. The mortality risk in nonoral cavity cancers was high in patients who received induction CT compared with those who received CCRT; the aHRs (95% CI) for head and neck cancer-related death were 2.91 (1.45–5.83) and 4.31 (3.38–5.50) in arms 2–3 and 3–3, respectively.

## DISCUSSION

CCRT is considered a curative treatment for locally advanced head and neck cancers. In 4 randomized trials, RT with concurrent CT was reported to be superior to RT alone;^11–13^ furthermore, induction CT has been associated with survival benefits, and it can be a valuable treatment option.^14–16^ The RTOG 9111 study evaluated RT alone versus concurrent RT and platinum CT versus induction CT (platinum and 5-FU) followed by definitive local RT (control arm); the 2-year laryngectomy-free survival rate was significantly higher for concomitant platinum-based induction CT and RT (66% vs 59%) than for RT alone (53%). Toxicity – particularly nausea, vomiting, and esophagitis – was the highest for concomitant cisplatin and RT. The combination of platinum CT and RT significantly improved laryngectomy-free survival (*P* = 0.01); however, no significant difference was observed in the overall survival.^11^ In the GORETC trial, the organ preservation rate with docetaxel-based induction was higher than that with platinum-based induction; however, the overall survival of these treatment modalities did not differ significantly.^3^ In the TAX 323 and 324 randomized trials, all patients received platinum- or 5-FU-based induction CT, and half of the patients also received docetaxel-based induction CT.^5,6^ Following 3 cycles of the induction CT, patients received RT with a weekly carboplatin dose or RT alone, and those with persistent disease underwent surgery as required. Although including docetaxel improves the overall survival,^5,6^ whether induction CT improves outcomes under full-dose chemoradiation remains unknown. In the PARADIGM trial, the effect of docetaxel, cisplatin, and 5-FU (TPF) followed by an adaptive strategy – low-dose carboplatin and radiation – was examined in patients responding well to treatments. Patients with poor responses and those in the control arm received full-dose platinum CT with a concomitant boost of radiation. The trial was slated to accrue 300 patients, but accrued only 150.^7^ The DECIDE trial compared split-course radiation with 5-FU and hydroxyurea alone and split-course radiation with 5-FU and hydroxyurea preceded by 2 TPF cycles. Docetaxel was continued during the CCRT in the experimental arm. This trial was originally slated to accrue 400 patients, but it ultimately accrued fewer than 300 patients. Moreover, the time to accrual was prolonged; however, the investigators believed that the extended time to accrual was warranted because the trial was adequately designed to address whether induction therapy improves outcomes.^8^ We believe that the data from these studies did not demonstrate the advantage of induction CT over current standard CCRT for head and neck cancers. Our study compared the survival of patients receiving docetaxel- or platinum-based induction CT administered before CCRT with that of patients receiving upfront CCRT alone.

Patients receiving platinum-based induction CT exhibited poor outcomes in all-cause and head and neck cancer-related death compared with those who received CCRT. These outcomes were consistent with those of a meta-analysis of CT in head and neck cancers.^17^ The meta-analysis was published almost a decade ago and included 63 randomized trials conducted over a 30-year period; it reviewed more than 10,000 patients, with a median follow-up of 6 years. The introduction of platinum-based CT to RT reduced the relative risk of death to 0.89. Concomitant approaches yielded the highest absolute benefit in the 5-year overall survival rate, which was 8%, higher than the 1% for adjuvant approaches and 2% for induction CT alone. The survival improved significantly with CCRT (*P* < 0.0001).^17^ An updated version of the aforementioned meta-analysis included 24 additional trials for approximately 18,000 patients, with a median follow-up of 5 years; in this more homogeneous population, an 8% absolute improvement (4.5% at 5 years in the updated analysis) in long-term survival was observed for combined modality approaches, specifically CCRT. No differences were observed in the survival benefits according to the anatomical sites – the oral cavity, oropharynx, hypopharynx, and larynx. Furthermore, no major differences were observed regarding T and N staging.^4^ In our study, platinum-based induction CT (arm 3) resulted in higher all-cause and head and neck cancer-related death, regardless of the anatomical site of the cancer, with the aHRs (95% CI) of 1.44 (1.36–1.5) and 1.47 (1.38–1.56), respectively (Table 2). The all-cause death aHR (95% CI) increased to 1.32 (1.23–1.42) in patients who completed induction CT with platinum + CCRT compared with those who received CCRT alone. Among head and neck cancer patients who could not tolerate induction CT with platinum + CCRT, the aHR (95% CI) for induction CT with platinum increased to 1.90 (1.71–2.11). For head and neck cancer-related death, patients who completed induction CT + CCRT had a higher aHR (1.36; 95% CI 1.26–1.46) compared with those who received CCRT alone. Among head and neck cancer patients who could not tolerate subsequent RT or CCRT after platinum-based induction CT, the aHR (95% CI) increased to 1.92 (1.71–2.16). The trend appeared to prioritize therapeutic benefits in the following order: CCRT, platinum + CCRT, platinum + RT, and induction CT with platinum alone with aHRs (95% CI) of 1 (0), 1.36 (1.26–1.46), 1.43 (1.26–1.46), and 1.92 (1.71–2.16), respectively. In our study, after adjustment for age, sex, CCI score, and cancer stage, platinum-based induction CT was an inferior therapy for head and neck cancer patients, regardless of whether the cancers were in the oral cavity (Figure 1A, B).

In Spanish randomized phase II and randomized phase III trials, the inclusion of docetaxel-based induction CT in CCRT showed no advantage; however, the incidence of adverse events increased.^18,19^ The 3-year overall (43% vs 55%) or progression-free (41% vs 50%) survival rates were not significantly different in the induction CT with docetaxel + CCRT and CCRT groups.^19^ PARADIGM and DECIDE were phase III trials that were closed to accrual and did not fulfill the predicted accrual targets. In both trials, docetaxel-based induction CT did not improve survival;^7,8^ however, a randomized phase II trial by Paccagnella et al^20^ demonstrated that adding docetaxel-based induction CT to CCRT increased the rate of complete radiological response compared with CCRT alone, and the trend favored progression-free and overall survival after docetaxel inclusion. Considering the core methodological flaws in these studies, the apparent failure or success of these trials does not justify the conclusion that induction CT lacks benefits. In our study, docetaxel-based induction CT resulted in poor survival and increased all-cause and head and neck cancer-related death. The aHRs (95% CI) for docetaxel-based induction CT (arm 2; Table 2) were 1.37 (1.22–1.53) and 1.29 (1.14–1.46) for all-cause and head and neck cancer-related death, respectively. In patients who completed docetaxel + CCRT therapy, disease-specific and overall survival for nonoral cavity cancers did not differ significantly between the docetaxel + CCRT and CCRT groups (Table 3), and these outcomes were similar to those of the aforementioned studies. Oropharyngeal cancer was the predominant cancer among nonoral cavity cancers included in the aforementioned studies, potentially because of the high incidence of human papillomavirus (HPV)-positive tumors among nonoral cavity cancers. Posner et al^21^ reported the overall and progression-free survival according to the HPV status in their TAX 324 study. According to overall and progression-free survival, HPV-positive patients had more favorable outcomes than HPV-negative patients did. The Southwest Oncology Group was scheduled to initiate a phase III trial addressing the role of HPV in oropharyngeal cancer; however, with the dissolution of the head and neck cancer committee in the Southwest Oncology Group, this trial did not proceed. In our study, docetaxel-based induction CT was an inferior therapy for head and neck cancer patients (Figure 1A, B), except for nonoral cavity cancer patients who completed docetaxel + CCRT. Our results suggest that outcomes of induction docetaxel + CCRT are adverse in nonoral cavity cancers. Additional studies or randomized clinical trials are necessary to validate this finding.

In Taiwan, more than 99% of head and neck cancers are squamous cell carcinomas, and more than 88% of head and neck cancer patients have a betel nut chewing habit.^22,23^ Betel nut chewers show higher incidences of local recurrence, distant metastasis, and secondary primary cancers as well as poorer disease-specific and overall survival than do nonchewers.^22^ In our study, more than 60% of the patients had oral cavity cancers and less than 15% of the patients had oropharyngeal cancers (Table 1). According to the anatomical site, the cancer occurrence and proportion differed considerably from those of other studies, because betel nut chewing is a common practice in Taiwan. In most studies on induction CT, oropharyngeal cancer (a nonoral cavity cancer) was the most prominent cancer, and on an average, more than 50% of head and neck cancer cases were of oropharyngeal cancer.^5–7,19,24^ This may explain why survival did not significantly differ between the docetaxel + CCRT and CCRT groups.^7,8,25^ In our study, among nonoral cavity cancer patients who tolerated docetaxel + CCRT, no significant difference in survival was evident between the docetaxel + CCRT and CCRT groups. In the future, data should be stratified according to the HPV status to illustrate the actual benefits of induction CT and enable suitable patient selection. We considered the ITT approach in our study; if head and neck cancer patients without distant metastasis received induction CT alone, then the patients would have dropped out after initial treatment because of an increase in the adverse events.^7,8,18,19^ The ITT approach provides unbiased comparisons among treatment groups and is used to avoid the effects of crossing over and dropping out, both of which may alter the outcomes of initial treatment assigned to the treatment groups. Overall, docetaxel- or platinum-based induction CT was inferior to CCRT in all-cause and head and neck-related death in our analysis (Figure 1A, B). No survival benefits were observed in oral cavity cancers in this study. Thus, platinum- or docetaxel-based induction CT is not recommended for oral cavity cancer patients.

In the present study, Table 1 shows that the CCRT group had a higher CCI score that those of the other groups, implying that it had more comorbidities. Thus, many deaths in the CCRT group can be attributed to comorbidities. The most favorable survival outcomes were observed in the CCRT group. Thus, we might have underestimated the risk of platinum- or docetaxel-based induction CT. Induction CT did not improve the survival benefits in head and neck cancer patients but increased the mortality risk. Dropout rates for platinum- and docetaxel-based induction CT were 9.94% and 21.90%, respectively (Table 2). The dropout rate was similar to that of the TAX 323, TAX 324, RTOG 9111, GORETC 2000-01, TREMPLIN, PARADIGM, and DECIDE trials.^3,5–8,11,26^ Overestimating the mortality risk of induction CT was difficult; the results were representative of actual outcomes because survival in patients receiving platinum- or docetaxel-based induction CT before CCRT was directly compared with that of patients receiving upfront CCRT alone. According to our literature review, this is the 1st study to directly compare the survival in patients receiving platinum- or docetaxel-based induction CT before CCRT with that of patients receiving upfront CCRT alone. The PARADIGM and DECIDE trials did not compare platinum-based induction CT with CCRT alone,^8,27^ but their CCRTs included cetuximab and docetaxel, respectively.

Docetaxel- or platinum-based induction CT + CCRT, induction CT + RT, or induction CT did not exhibit any advantage compared with CCRT alone in patients with head and neck cancers without distant metastasis (Figure 1A, B). The only feasible modality of induction CT was induction docetaxel + CCRT for nonoral cavity cancers because all-cause and head and neck cancer-related mortality risk were equal for the docetaxel + CCRT and CCRT groups. Thus, the current study provides data that can be elaborated on to form detailed hypotheses for future studies and illustrates that along with docetaxel-based induction CT for nonoral cavity cancers, an HPV test is warranted to improve patient survival.

The following are the limitations of our study. The actual toxicity induced by induction CT and the organ preservation rate could not be determined. HPV test data were not recorded in the included databases; hence, the effect of induction CT on HPV-positive or -negative patients could not be examined. All investigated head and neck cancer patients were from an Asian population, and differences in susceptibility among races remain undetermined; hence, our results should be cautiously extrapolated to non-Asian populations. A large-scale randomized trial with a suitable regimen administered to carefully selected patients and comparing standard approaches is essential for obtaining crucial information regarding population specificity and disease occurrence. Furthermore, diagnoses of all comorbidities were completely dependent on ICD-9-CM codes. Nevertheless, the Taiwan NHI Bureau randomly reviews charts and interviews patients to verify the accuracy of the diagnoses. Hospitals with outlier charges or practices may undergo an audit and subsequently receive heavy penalties if malpractice or discrepancies are discovered. Finally, the databases contain no information regarding tobacco use, alcohol consumption, dietary habits, socioeconomic status, and body mass index, all of which may be mortality risk factors. The differences in all-cause and head and neck cancer-related death in the docetaxel + RT and CCRT groups did not differ significantly because the sample size was small (65 patients). However, a trend of all-cause mortality risk was observed, and the aHR was 1.35 (95% CI 1.00–1.82, *P* = 0.052). Thus, docetaxel- or platinum-based induction CT was an independent risk factor for death (Figure 1A, B). However, given the magnitude and statistical significance of the observed effects in this study, these limitations are unlikely to have affected our conclusions.

Our cohort study showed that induction CT with docetaxel or platinum did not improve survival and resulted in higher all-cause mortality and head and neck cancer-related risk than did CCRT. No survival benefit or increase in mortality was observed in oral cavity cancer patients; therefore, platinum- or docetaxel-based induction CT is not recommended for these patients.

## Supplementary Material

Supplemental Digital Content
