# Supplementary material for: Outcomes of Induction Chemotherapy for Head and Neck Cancer Patients: A Combined Study of Two National Cohorts in Taiwan
Source: Medicine (Baltimore). 2016 Feb 18;95(7):e2845. doi: 10.1097/MD.0000000000002845 (PMC4998647; doi:10.1097/MD.0000000000002845)
Supplement: Supplemental Digital Content [file medi-95-e2845-s001.docx]

**Supplemental table 1.** Power analysis of current study

| Induction chemotherapy | HR | Sample size | P_E_ | P_I_ | Power |
| --- | --- | --- | --- | --- | --- |
| Docetaxel induction | 1.32 | 8489 | 0.6103 | 0.0593 | 0.9971 |
| Platinum induction | 1.42 | 10218 | 0.6103 | 0.2184 | >0.9999 |

P_E_: overall probability of event

P_I_: proportion of sample in the specific induction chemotherapy group
